# Prevalence of oral manifestations in patients with lupus erythematosus in a sample of the Egyptian population: a hospital based cross-sectional study

**DOI:** 10.12688/f1000research.55332.4

**Published:** 2022-06-06

**Authors:** Hager Moustafa Saeed, Eman Mohammad Amr, Alshaimaa Rezk Lotfy Rezk, Wesam Abd Elmoneim

**Affiliations:** 1 Department of Oral Medicine and Periodontology, Faculty of Dentistry, Cairo University, Cairo, Egypt; 2 Department of Oral Medicine and Periodontology, Cairo University, Cairo, 11562, Egypt; 3 Department of Internal Medicine, Cairo University, Cairo, 11562, Egypt

**Keywords:** lupus erythematosus, oral manifestations, precancerous oral lesions, SLE

## Abstract

**Background: **Several systemic diseases manifest themselves in the oral cavity. Dentists who are unaware of these lesions will possibly miss them. This cross-sectional study aimed to assess the prevalence of oral manifestations in patients with LE in a sample of the Egyptian population.

**Methods: **The present cross-sectional study was performed on 189 patients attending the Internal Medicine Department, Rheumatology Clinic in EL Qasr El Ainy Hospital, Cairo University. Every patient was examined clinically after completing a questionnaire. Patients’ medical records were evaluated.

The oral manifestations were assessed according to the WHO guide to physical examination of the oral cavity and classified according to their morphologic aspects and localization.

**Results: **Out of 189 patients, there were 182 females (96.3%) and seven males (3.7%). The prevalence of oral lesions in SLE patients was 55.6%. The most affected site was the tongue 25.7%. The most common clinical aspect was patches, 53%. About 77.1% of the lesions were asymptomatic.

**Conclusions: **The present study emphasizes the importance of early diagnosis of oral lesions to recognize patients with SLE as the WHO considers oral manifestations of SLE a widespread state. Also, the implementation of oral hygiene measures to improve patients’ nutritional state and health-related quality of life is recommended.

## Introduction

Lupus erythematosus (LE) is an autoimmune disease subdivided into a cutaneous and systemic forms. The prevalence of mucosal involvement in LE patients is debatable. ^1^ There is a wide range of the prevalence of mucosal involvement based on population. ^2^^–^^4^ The mucosal involvement of LE ranges from 9–45% in systemic lupus erythematosus (SLE) and 3–20% in cutaneous lupus erythematosus (CLE). ^1^

The morphologic aspects of the oral lesions presented in SLE, presented in varied clinical aspects, a red macula or plaque, ulcerations surrounded or not by white irradiating striae to a white plaque on a pigmented mucosa. ^5^

Clinical features differed according to the anatomical location. Lesions of the hard palate were red maculae or plaque, in contrast, white lesions (plaque and lichen-like striae) were found only in the buccal mucosa. Lesions of the lips ranged from red plaques or ulcers. However, a white plaque on pigmented mucosa was also reported. ^6^

In descending order, locations frequently affected were the buccal mucosa, hard palate, and lower lips. Some patients had lesions simultaneously with more than one oral site. While in a more recent study, it was reported that the most commonest site of oral findings was on the hard palate. Other sites included the labial mucosa, buccal mucosa, gingiva, and alveolar ridge. ^1^

As mentioned in the WHO digital manual for the early diagnosis of oral neoplasia (2008), several systemic diseases manifest themselves in the oral cavity. These lesions can precede the symptoms and signs of systemic disease or can coexist with it and dentists who are unaware of these lesions will possibly miss them. ^7^

According to WHO guides for screening programs (2009), ^8^ most programs are selective and target a subset of the population who are considered to be at the highest risk. ^9^ Consequently, the present study assessed the prevalence of oral manifestations among a sample of Egyptian patients recently diagnosed with lupus erythematosus as they are considered to be at a high risk of developing oral precancerous lesions.

## Methods

The present cross-sectional study was performed to assess the prevalence of oral manifestations in patients with lupus erythematosus in a sample of the Egyptian population. The study was held in the Internal Medicine Department, Rheumatology Clinic in EL Qasr El Ainy Hospital, Cairo University. Hospital data collection started in March 2019 until March 2020.

**Inclusion criteria:** Patients are immediately diagnosed with systemic lupus erythematosus based on American College of Rheumatology (ACR) criteria. The age of the patients was 14–70 years old. Both genders were included. Cigarette smoking patients were included. ^10^

**Exclusion criteria:** Patients suffering from any other systemic diseases. Patients on drug therapy which may cause oral mucosal manifestations. ^11^

For each eligible participant, a full history was obtained through an interview between the investigator and the patient. Demographical data were collected. ^12^ All participants were asked to sign a study-related informed consent. The clinical examination of the oral manifestation was recorded by conventional oral examination (COE) according to the WHO digital manual for physical examination of the oral cavity. SLE patients who had an oral manifestation as present and SLE patients without oral manifestation as absent. The oral manifestations were interpreted according to their clinical aspects and their sites in the oral cavity. ^12^^,^^13^ Cigarette smoking patients were assessed. ^14^

The primary outcome was the prevalence of intraoral manifestations. Selection bias was minimized by enrolling the participants in the study in consecutive order of them entering the clinic. Non-respondent bias was minimized by explaining to the participants the aim of the study and their importance and role in the study. Incomplete records were excluded from statistical analysis with the cause of an incomplete record reported.

Ethical approval for the questionnaire and methodology was approved by the Ethics Committee of the Faculty of Dentistry, Cairo University, Cairo, Egypt (approval number: 19/5/6).

Sampling was conducted continuously, and the sample size was considered 189 patients with lupus erythematosus with a 95% confidence level, 5% margin of error, and 7.1 maximum deviation of the sample rate. The sample size was calculated using Stats Direct statistical software (version 3.1.17) (An open-access alternative that can provide an equivalent function is the R stats package (RRID:SCR_001905)). Qualitative data were presented as frequencies and percentages. Quantitative data were presented as mean, standard deviation (SD), and 95% confidence interval (95% CI) for the mean values. For qualitative data, Fisher’s Exact Test was used for comparisons regarding qualitative variables. Quantitative data were explored for normality by checking the distribution of data and using tests of normality (Kolmogorov-Smirnov and Shapiro-Wilk tests). Age data showed a parametric distribution. The Student’s t-test was used to compare patients without and with oral lesions. The significance level was set at *P* ≤ 0.05. Statistical analysis was performed with IBM SPSS Statistics for Windows, Version 23.0. (Armonk, NY: IBM Corp) (RRID:SCR_019096) (An open-access alternative that can provide an equivalent function is the R stats package (RRID:SCR_001905)).

## Results

A total of 189 patients with LE were included in the study. All the sampled patients met the ACR criteria for diagnosis of SLE. CLE wasn’t found among the sampled patients.

The mean (SD) values for age were 30.5 (9.7%). Only four patients (2.1%) were smokers. Four women (2.2%) were pregnant.

In this study, the prevalence of oral lesions among SLE patients was 55.6% (105/189 patients). 182 females (96.3%) and 7 males (3.7%). This showed a non-significant relationship in terms of gender in the prevalence of oral manifestations ( *P*-value = 0.465, Effect size = 0.769). There was no statistically significant difference between mean age values in patients with and without oral lesions ( *P*-value = 0.210, Effect size = 0.187). There was no significant relationship between smoking and non-smoking patients. Patient details are summarized in Table 1 and are shown in the underlying data. ^15^

**Table 1.** Descriptive statistics, results of Fisher’s Exact test, and Student’s t-test for comparison between patients with and without oral lesions.

		No oral lesion (n = 84)	Oral lesion (n = 105)	*P*-value	*Effect size*
Gender [n, (%)]	Male	2 (2.4%)	5 (4.8%)	0.465	*OR* = 0.769
	Female	82 (97.6%)	100 (95.2%)		
Age [Mean, (SD), 95% CI]		31.5 (9.7), 29.4–33.7	29.7 (9.7), 27.8–31.6	0.210	*d* = 0.187
Smoking [no. (%)]	Smoker	2 (2.4%)	2 (1.9%)	1.000	*OR* = 0.016
	Non-smoker	82 (97.6%)	103 (98.1%)		

* Significant at *P* ≤ 0.05.

**Figure 1.**
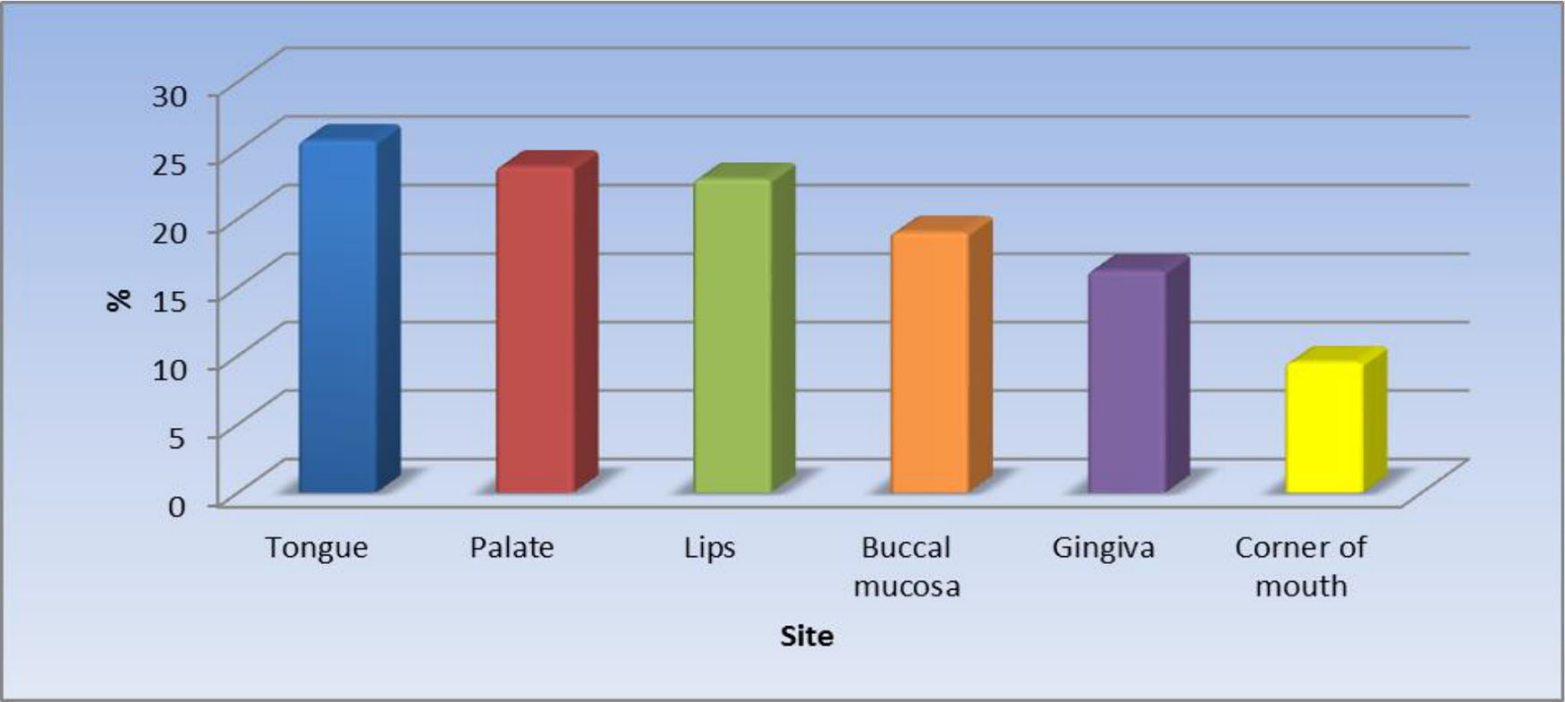
Clinical sites of the oral lesions (n = 105).

**Figure 2.**
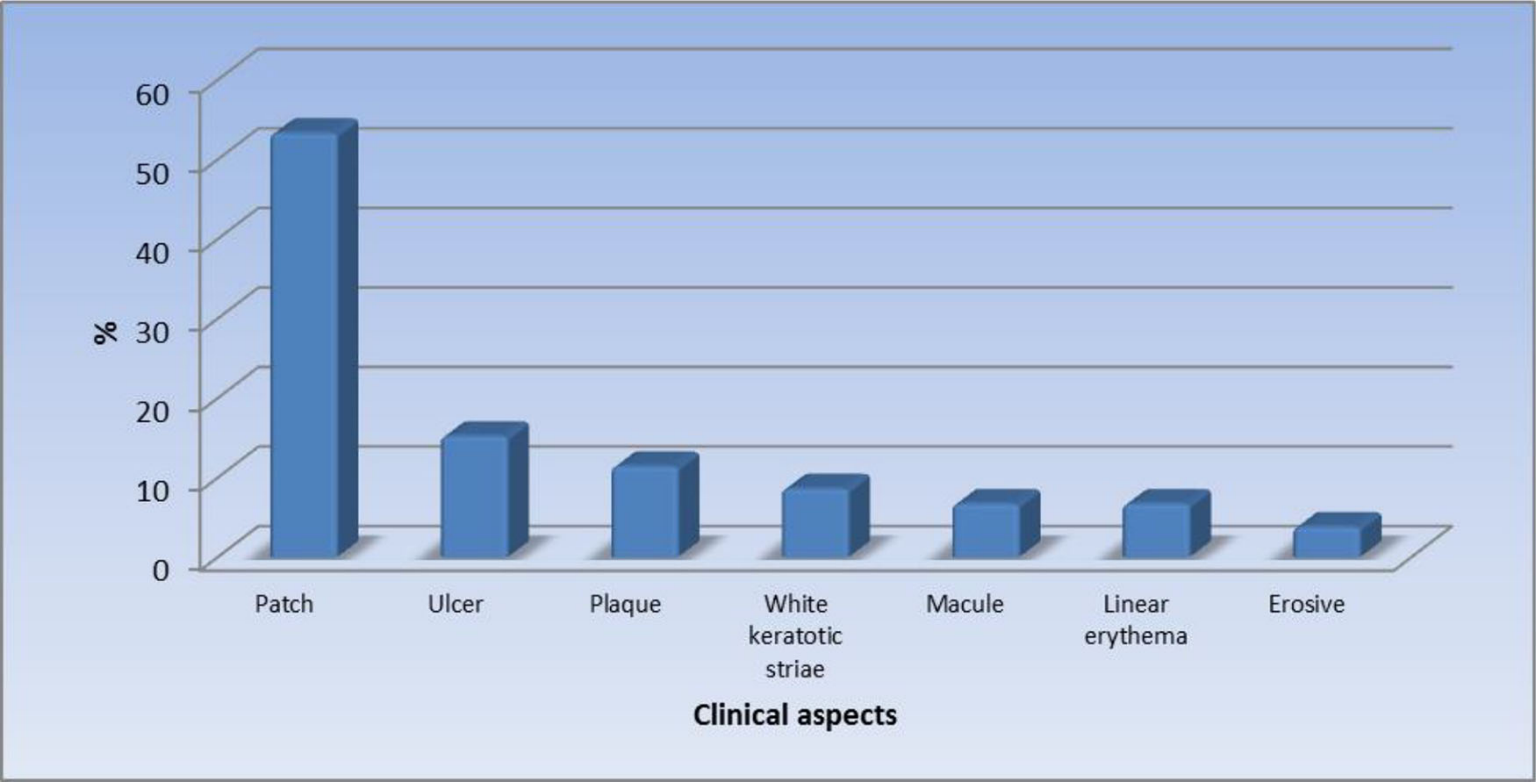
Clinical aspects of the oral lesions (n = 105).

Of the 105 patients (55.6%) with oral lesions, the most affected site was the tongue 25.7%. Figure 1 displays the site of the oral lesions in descending order. The most common clinical aspect was patches, 53%. Figure 2 displays the clinical aspect of the oral lesions in descending order. Twenty-four patients (22.9%) had a burning sensation while 81 patients (77.1%) were asymptomatic.

Table 2 shows the difference in the prevalence of oral manifestations in SLE patients among regions and countries.

**Table 2.** The prevalence of oral manifestations in different countries.

Geographic data	North Africa		Middle East and Asia								
Parameter	Our series	Tunisia ^25^	Saudi Arabia ^26^	Qatar ^21^	UAE ^27^	Kuwait ^28^	Lebanon ^29^	Iraq ^24^	Iran ^16^	China ^30^	Pakistan ^31^
Number of patients	189	749	624	77	110	108	100	50	188	709	196
Mean age	30.5 (9.7)	30.66 (11.4)	25.3 (63.6)	38.3 ± 10.6	28.9 (0)	31.5	25	-	33	30.1 (12.1)	31
Sex	26:11:00	9.6:1	9.8:1	9.5:1.	20.5:1	10:01	6.1:1	17:01	162/26	9.3:1	7.2:1
Malar rash	-	68.7	47.9	-	62	43	43	-	-	56	29
Oral manifestations	55.6%	-	-	88.10%	-	-	-	54%	54%	-	-
Oral ulcers	15.20%	23.30%	39.10%	2.40%	23.90%	33%	40%	72%	28.10%	35%	19.70%

Geographic data	Europe			South Africa	South America				USA	
Parameter	Ireland ^17^	Turkey ^32^	Europe ^33^	South Africa ^34^	Argentina ^18^	Venezuela ^6^	Brazil ^1^	Latin America ^35^	USA ^36^	
Number of patients	42	428	1000	111	150	90	46	1214	256	
Mean age	48	40.3 (12.4)	29	35	39.1 (13.7)	36.39 (8.42)	41.8 years.	28 (12)	39.9 (14.8)	
Sex	37/42	13.8:1	10:01	12.8:1	(139/150)	10.25:1	2.83:1	8.8:1	9.6:1	
Malar rash	-	-	58	55	45.30%	-	-	61.3	38	
Oral manifestations	50%	-	-	-	97%	11.10%	100%	-	-	
Oral ulcers	23%	38.80%	24%	33%	7%	-	21%	41.70%	17%	

## Discussion

The current descriptive study assessed the prevalence of oral manifestation among SLE patients in Egypt.

The present study was conducted on 189 patients: 182 females (96.3%) and seven males (3.7%), and this indicated that SLE is more prevalent in Egyptian females than in males. This finding agreed with López-Labady *et al*., ^6^ Khatibi *et al*., ^16^ Ali *et al*., ^17^ as well as Barrio-Díaz *et al*., ^18^ who also found that the majority of SLE patients were female.

Despite the variation in sample size between all studies, males were less affected by oral manifestations than females. ^12^ There was systemic involvement in all the sampled patients. CLE patients weren’t found in the sampled population. This explains the fact that CLE may be part of the spectrum of SLE or be an entity alone with no systemic features. ^19^

There was no statistically significant association between the prevalence of gender and oral lesions. Moreover, there was no significant difference between mean age values in patients with and without oral lesions. These findings agreed with Khatibi *et al*., (2012). ^16^ There was no statistically significant association between smoking and oral manifestations. This agreed with a study by Bourré-Tessier *et al*., ^20^ who reported that there was no clear association between smoking and the presence of mucosal ulcers or malar rash.

The present study showed that the prevalence of oral manifestations was 55.6% (105/189 patients). In a study conducted in Iran, 102 (54.3%) out of 188 patients had oral lesions, while 86 (45.7%) had none. ^16^ In addition to that, a study conducted in Ireland showed that 50% of patients had positive oral findings. ^17^ In Saudi Arabia it was found that mucocutaneous lesions including oral ulcers were reported in 72% of 46 SLE patients. ^21^ Also, De Rossi *et al*., in 1998, found the prevalence of oral manifestations ranged from 81.3 to 87.5%. ^16^ The highest prevalence was reached at 97% in an Argentinian study. ^17^ On the other hand, a lower prevalence was shown in a Venezuelan study, ^6^ which reported that of the 90 patients diagnosed with SLE only 10 patients (11.1%) showed oral mucosal lesions. Collectively, the higher prevalence of oral manifestations in SLE is probably because all tissues are potentially affected as a result of the disease course. ^1^

The results of the current study revealed that the most affected site was the tongue (25.7%) in just over one-quarter of the patients followed by the palate, lips, buccal mucosa, the gingiva, and the least affected site was the corner of the mouth. Khatibi *et al*., in 2012, revealed that the sites most commonly affected by oral lesions were the buccal mucosa and the lips. ^6^ A Brazilian study reported that the more frequently affected sites were the buccal mucosa than the hard palate and lower lips. ^1^ While another study found that the most commonest site was the hard palate. ^17^ This variation may be attributed to dissimilarity in the exclusion and inclusion criteria of these studies.

The second most frequently affected site for oral manifestations in this study was the palate and this agreed with a previous study conducted in Brasil. ^1^ In third place were the lips; the lower lips were more often affected than the upper lips. This may be attributed to the fact that the lower lips are more exposed to sunlight than the upper lips and to the biological mechanisms of ultraviolet rays (UVR), which induce lupus flare. ^22^

In our study, patches were reported as the most significant morphologic feature (53.3%). This was followed by ulcers (15.2%), plaques (11.4%), white keratotic striae (8.6%), macules (6.7%), and linear erythema (6.7%), and the least common clinical feature was erosive lesions in 3.8% of the patients.

Lourenco *et al*., (2007) reported that oral lesions presented in different clinical aspects, ranging from classic plaques accompanied by central erythema enclosed by a white rim with radiating keratotic striae to a white plaque on a pigmented mucosa and finally to bullous lesions. ^1^ Menzies *et al*., reported that SLE lesions varied from striated/reticular white patches, erosions, and ulceration to homogenous white patches. ^17^ Recently, Barrio *et al*., reported that oral lesions were classified into erythematous patches, honeycomb plaques, lupus cheilitis, chronic plaques, oral discoid lesions, LP-like lesions, keratotic lesions, ulcerative plaques, oral ulcers, pebbly red areas, purpuric lesions, erythema, and diffuse palatal petechial erythema. ^18^

The results of the current study revealed that the clinical appearance of the patches varied from one patient to another. Round erythematous patches were reported in 35.2% of the lesions. These patches were painless and would bleed on palpation while scaly erythematous patches were observed in 16.2% of the lesions. A scaly white patch was reported in 1.9% of the patients, particularly on the lips, these scales were crusted and thick. Barrio *et al*., (2020) reported that erythematous patches are considered clinical descriptions of oral lupus lesions. ^18^ Nico *et al*., ^1^ reported that SLE oral lesions manifested as oval non-scarring patches with variable degrees of erosion. ^1^

The second most significant clinical feature was found to be the ulcer. Ulcers were reported in 15.3% of cases, ranging from ulcers surrounded by a red halo, painless ulcers surrounded by white radiating striae, ulcers surrounded by red radiating striae associated with burning sensation, and round erythematous hemorrhaging ulcers.

Meyer *et al*., and Ranginwala *et al*. found that oral ulcers are present in 19% of cases in both of their studies. ^23^ While Khatibi *et al*., (2012) and Menzies *et al*., (2018) found that 28.1% and 23.8% of patients showed oral ulcers respectively. ^16^ Barrio *et al*., (2020) found that oral ulcers were present in 11 of 150 patients with systemic lupus (7%). ^18^ On the other hand, Ali *et al*. (2012) reported that oral ulcers were present in 72% of patients. ^24^

The third clinical picture in our study was the plaque. Plaques were reported in 11.4% of the lesions. The clinical appearance ranged from painless red plaques to painful erosive plaques. Lourenço *et al*., (2007), found that the lesions of the hard palate were red maculae or plaque. In contrast, white lesions were found only in the buccal mucosa. ^1^ Barrio *et al*. reported that honeycomb plaques on the palate are only present in systemic lupus patients. ^18^ A white plaque on pigmented mucosa was reported by López *et al*. ^6^ Also, Lourenço *et al*., reported four cases of classic plaques with central erythema from 46 patients (8.6%). ^1^

In the current study, painless white keratotic striae came in fourth place at 8.6%. Buccal mucosa was the most affected by white keratotic striae followed by the gingiva. These findings agreed with Lourenço *et al*., who reported that white lesions (plaque and LP-like striae) were found only in the buccal mucosa. ^1^

The results of the present study revealed that single and cluster macules were reported in 6.7% of the cases. These red macules were painless, and the palate showed the highest prevalence of macules followed by the gingiva. This was in accordance with López *et al*., who also reported the presence of red maculae on the hard palate. ^6^ Barrio *et al*., reported that high activity of the SLE was associated with red macules on the soft palate and brown-pigmented macules on the lower gingiva. ^18^

In the current study, linear erythema was reported in 6.7% of cases. It was noticed on the gingiva and palate. Similarly, Nico *et al*., 2008 reported that linear erythema and keratosis were observed on the upper palatal gingiva in the patient. ^1^

Finally, erosive lesions were observed in 3.8% of the cases in the present study. These lesions showed no statistically significant association with a particular oral site. A Brazilian study reported erosive lesions on the lips and buccal mucosa. ^1^ Also, erosive and keratotic lesions on the left buccal mucosa were presented in a case report by Nico *et al*., (2008). ^1^

This study is a descriptive study to assess the prevalence of oral manifestation in systemic lupus patients not include the clinical manifestation, drug treatment of patients, and clinical associations/statistical analysis.

## Recommendations

Further studies should be conducted in other regions with larger sample size and at different time intervals to broaden these findings. Also, additional research could highlight the impact of race, ethnicity, and genetics on the prevalence of oral manifestations of the disease.

## Conclusion

The present study emphasizes the importance of early diagnosis of oral lesions in patients recognized with SLE as the WHO considers oral manifestations of SLE as a widespread state. It is also required to implement oral hygiene measures and to improve patients’ health-related quality of life. Further studies are suggested to be conducted on larger sample size and at different intervals.

## Data availability

### Underlying data

Dryad: Underlying data for ‘Prevalence of oral manifestations in patients with lupus erythematosus in a sample of the Egyptian population: a hospital-based cross-sectional study’, https://doi.org/10.5061/dryad.wstqjq2mv. ^15^

This project contains the following underlying data:

• Data file 1: Prevalence of oral manifestations in SLE patients.xlsx
• Data file 2: Read_me.txt

Data are available under the terms of the Creative Commons Zero “No rights reserved” data waiver (CC0 1.0 Universal Public domain dedication).

## Consent

All participants gave their informed consent to the interviewer verbally, using the telephone interview as a format for data collection. In addition, a link to the consent form was sent electronically requesting written consent for publication of the patients’ details.
