# Supplementary material for: Prevalence of oral manifestations in patients with lupus erythematosus in a sample of the Egyptian population: a hospital based cross-sectional study
Source: F1000Res. 2022 Jun 6;10:969. Originally published 2021 Sep 27. [Version 4] doi: 10.12688/f1000research.55332.4 (PMC9178282; doi:10.12688/f1000research.55332.4)
Supplement: Peer review history

# Peer review: Reviewer response for version 4

**DOI:** 10.5256/f1000research.134585.r139745

**Published:** 2022-06-08

**Authors:** Ljudmila Stojanovich

**Affiliations:** 1 University Medical Center of Belgrade University, Belgrade, Serbia

I believe that now this manuscript can be accepted and indexed in a journal.

Is the work clearly and accurately presented and does it cite the current literature?

Partly

If applicable, is the statistical analysis and its interpretation appropriate?

Yes

Are all the source data underlying the results available to ensure full reproducibility?

Partly

Is the study design appropriate and is the work technically sound?

Partly

Are the conclusions drawn adequately supported by the results?

Partly

Are sufficient details of methods and analysis provided to allow replication by others?

Partly

Reviewer Expertise:

NA

I confirm that I have read this submission and believe that I have an appropriate level of expertise to confirm that it is of an acceptable scientific standard.

# Peer review: Reviewer response for version 3

**DOI:** 10.5256/f1000research.122370.r135160

**Published:** 2022-05-13

**Authors:** Ljudmila Stojanovich

**Affiliations:** 1 University Medical Center of Belgrade University, Belgrade, Serbia

The manuscript “Prevalence of oral manifestations in patients with lupus erythematosus in a sample of the Egyptian population: a hospital based cross-sectional study” by Saeed HM, *et a*l. evaluated the presence of prevalence of oral manifestations in SLE patients.

 In the current study, the authors conducted a nation cohort of the Egyptian population.

 Strength:

- This study comprises important information for the Egyptian population.

Limitation:

- There is nothing new except the Egyptian population.

It is well known that oral manifestations are very often present in SLE, and lip ulcers are also a standard symptom of the disease (American College of Rheumatology).

 Most importantly, there is no correlation between oral manifestations and activity of the disease/SLE, according to any recognized criteria of activity (SLEDAI), and there is no clear connection with therapeutic treatment. The suggestion is to state clearly, preferably in tabular form, which manifestations were seen in the patient with which drug, and the dose of the drug.

 Some tables are completely unnecessary, e.g., table number 1.

 There are no data on how many patients had completely recovered teeth. There is no information on whether the patients had any infection? It is necessary to include “rare infections/ or opportunistic infections.

 However, the major disadvantage of the study is the fact that the results do not bring forth novel data of clinical and scientific importance.

 At the same time, the presentation of the cohort is not clear. It is not specified if other criteria manifestations were present concomitantly with skin manifestations. Were these manifestations present simultaneously with the other events? Such as cutaneous skin, changes in the CNS, lupus nephritis and others? What is the rationale behind this?

 The suggestion to add a few more published papers to the reference, for example: *Stojanovich L, Djokovic A, Stanisavljevic N, Zdravkovic M.* *The cutaneous manifestations are significantly related to cerebrovascular in a Serbian cohort of patients with Hughes syndrome.* *Lupus. 2018, Vol.27, 5: 858-863.* ^1^

 The authors’ conclusion is not fully supported in this data

 The methodology is of low quality I thus suggest that the work be accepted after a major revision.

Is the work clearly and accurately presented and does it cite the current literature?

Partly

If applicable, is the statistical analysis and its interpretation appropriate?

Yes

Are all the source data underlying the results available to ensure full reproducibility?

Partly

Is the study design appropriate and is the work technically sound?

Partly

Are the conclusions drawn adequately supported by the results?

Partly

Are sufficient details of methods and analysis provided to allow replication by others?

Partly

Reviewer Expertise:

Autoimmune disease

I confirm that I have read this submission and believe that I have an appropriate level of expertise to confirm that it is of an acceptable scientific standard, however I have significant reservations, as outlined above.

## References

[1] : The cutaneous manifestations are significantly related to cerebrovascular in a Serbian cohort of patients with Hughes syndrome. *Lupus* .2018;27(5) : 10.1177/0961203317751065 858-863 10.1177/0961203317751065 29301476

# Response

**Published:** 2022-05-29

**Authors:** Hager Saeed

**Affiliations:** october 6 university, Egypt

Dear Ljudmila,

 Please accept our sincere gratitude for your insightful input, and we eagerly await the opportunity to learn from you. If you don't mind, we'll demonstrate some aspects to clear up your doubts.

 The study only included patients who were diagnosed immediately according to ACR criteria. All of the patients in Clinic One had never used lupus medication before. The research was carried out at clinic number one, which is solely for new patients.

 According to the inclusion criteria, only patients who were diagnosed immediately and had never taken lupus medication before were included.

 Table 1 displays the demographic data of the patients, however, table 2 will take its place.

 The primary outcome was the prevalence of intraoral manifestations in **immediately diagnosed and had not previously received any lupus medication. The study design is cross-sectional study.**

**We didn’t perform any follow-up for the patients.**

 This reference was used in

 •    Khatibi M, Shakoorpour AH, Jahromi ZM, Ahmadzadeh A. The prevalence of oral mucosal lesions and related factors in 188 patients with systemic lupus erythematosus. Lupus. 2012 Oct;21(12):1312–5.

 •    Hammoudeh M, Al-momani A, Sarakbi H, Chandra P, Hammoudeh S. Oral Manifestations of Systemic Lupus Erythematosus Patients in Qatar: A Pilot Study. International Journal of Rheumatology. 2018;2018:1-7. doi:10.1155/2018/6052326

 •    Hadidi KT El, Medhat BM, Baki NMA, Kafy HA, Abdelrahaman W, Yousri AY. Characteristics of systemic lupus erythematosus in a sample of the Egyptian population : a retrospective cohort of 1109 patients from a single center. 2018;(December 2017):1–9.

 The conclusion was based on the WHO digital manual for the early diagnosis of oral neoplasia (2008)

 you will find it in 

 https://screening.iarc.fr/atlasoral_list.php?cat=A7&lang=1

# Peer review: Reviewer response for version 3

**DOI:** 10.5256/f1000research.122370.r127698

**Published:** 2022-03-22

**Authors:** Syahrul Sazliyana Shaharir

**Affiliations:** 1 Department of Internal Medicine, Rheumatology Unit, Universiti Kebangsaan Malaysia Medical Center, Kuala Lumpur, Malaysia

Authors have addressed all the comments

Is the work clearly and accurately presented and does it cite the current literature?

No

If applicable, is the statistical analysis and its interpretation appropriate?

Yes

Are all the source data underlying the results available to ensure full reproducibility?

Partly

Is the study design appropriate and is the work technically sound?

Yes

Are the conclusions drawn adequately supported by the results?

Yes

Are sufficient details of methods and analysis provided to allow replication by others?

No

Reviewer Expertise:

SLE

I confirm that I have read this submission and believe that I have an appropriate level of expertise to confirm that it is of an acceptable scientific standard.

# Peer review: Reviewer response for version 2

**DOI:** 10.5256/f1000research.120062.r122129

**Published:** 2022-02-15

**Authors:** Syahrul Sazliyana Shaharir

**Affiliations:** 1 Department of Internal Medicine, Rheumatology Unit, Universiti Kebangsaan Malaysia Medical Center, Kuala Lumpur, Malaysia

1. Suggest to use the standard abbreviation of Systemic Lupus Erythematosus (SLE) in the manuscript as all the patients fulfilled the ACR classification criteria for SLE. 
2. Introduction- should mainly focus on SLE and elaborate more on the prevalence, type and location of oral lesions from other literature. It was stated that oral lesion in SLE is associated with malignancy but need citation and be specific on what type of lesion and the location
3. Results: To include a summary on the system/ clinical manifestation and drug treatment of patients who were included in the study, if this information is available. If the data is not available, acknowledge these limitations in the discussion and emphasize that this study was mainly a descriptive study and lack of clinical associations/statistical analysis.

Is the work clearly and accurately presented and does it cite the current literature?

No

If applicable, is the statistical analysis and its interpretation appropriate?

Yes

Are all the source data underlying the results available to ensure full reproducibility?

Partly

Is the study design appropriate and is the work technically sound?

Yes

Are the conclusions drawn adequately supported by the results?

Yes

Are sufficient details of methods and analysis provided to allow replication by others?

No

Reviewer Expertise:

SLE

I confirm that I have read this submission and believe that I have an appropriate level of expertise to confirm that it is of an acceptable scientific standard, however I have significant reservations, as outlined above.

# Response

**Published:** 2022-03-11

**Authors:** Hager Saeed

**Affiliations:** october 6 university, Egypt

Dear Syahrul,

 Thank you for your helpful advice. I gained a lot of knowledge from your reports.

 1.    Suggest using the standard abbreviation of Systemic Lupus Erythematosus (SLE) in the manuscript as all the patients fulfilled the ACR classification criteria for SLE. 

 Thanks for your suggestion, We changed the abbreviation to SLE

 2.    Introduction- should mainly focus on SLE and elaborate more on the prevalence, type and location of oral lesions from other literature. It was stated that oral lesion in SLE is associated with malignancy but need citation and be specific on what type of lesion and the location

 Thanks for your counsel. The reference of the oral lesion in SLE is associated with malignancy was cited in WHO digital manual digital manual for the early diagnosis of oral neoplasia. 

 https://screening.iarc.fr/atlasoral_list.php?cat=A7&lang=1

 3.    Results: To include a summary on the system/ clinical manifestation and drug treatment of patients who were included in the study, if this information is available. If the data is not available, acknowledge these limitations in the discussion and emphasize that this study was mainly a descriptive study and lack of clinical associations/statistical analysis.

 Thanks for your comment I added your comment in the discussion.

# Peer review: Reviewer response for version 1

**DOI:** 10.5256/f1000research.58897.r101743

**Published:** 2022-01-05

**Authors:** Laura B. Lewandowski

**Affiliations:** 1 National Institute of Arthritis and Musculoskeletal and Skin Diseases, National Institutes of Health, Bethesda, MD, USA

I think this paper has some merit - there is data here on one of the most common clinical manifestations in a specific regional cohort.

 However, the current version lacks focus and organization, and I cannot recommend indexing in the current format.

 The authors should consider a revision which focuses on the clinical description of oral lesions, both type and location, in their cohort. They should compare overall demographics and clinical features, and specific oral manifestations in their cohort to the published literature. Then they should state any unique features of their cohort in regards to oral lesions in SLE.

 Some of the introduction and discussion needs major reorganization and removal of statements that do not have evidence.

 Lupus erythematosus (LE) is an autoimmune disease subdivided into a cutaneous and a systemic form - It seems that the authors only included SLE patients based on 1997 ACR Criteria. If so, this distinction is distracting from the focus of the paper.

**Introduction:**

- "The prevalence of mucosal involvement in LE patients is debatable." - There are multiple reports on mucosal involvement in SLE. Authors should state there is a range based on population and cite the following: ^1^^,^^2^^,^^3^
- "The WHO considers the oral manifestations of LE as a widespread state associated with a significantly increased risk of cancer.” - This is not validated in the SLE literature. The citation listed here is a book review and does not support this claim.
- Inclusion criteria: Patients recently diagnosed with lupus erythematosus based on American College of Rheumatology (ACR) criteria. How did the authors define a recent diagnosis?
- Patients with an anti-nuclear antibody (ANA) positive test were only included in the study.
- Exclusion criteria - Patients who had received any previous therapy for lupus erythematosus. Authors should state treatment of naïve patients in the inclusion criteria.
- "Patients on drug therapy who may have oral mucosal manifestations, which eliminate all the potential confounders" - untrue. Reduced confounding due to medication effect.
- "The diagnosis of oral candidiasis was made by curd-like patches on the tongue or other oral mucosal surfaces, the presence of classic pseudomembranous lesions characterized by a creamy white pseudomembrane" - was this confirmed by any culture or biopsy?

**Results:**

- Table 1 should include an overall demographic data for all participants- age, sex, smoking, presence of oral lesions.
- Table 1 should be Table 2.
- Figure 1 - would change to anatomical sites of oral lesions. How did the authors standardize the bounds of each lesion?
- Figure 3: I am a bit confused about the inclusion of the data on the skin manifestations. Unless the authors have a specific hypothesis they would like to explore with this data, it seems out of place in this paper on oral lesions in SLE.
- "The sampled population was classified into two groups. LE patients who had an oral manifestation as true positive oral lesions (TP) and LE patients without oral manifestation as true negative oral lesions (TN)." - I think this language is confusing, as they do not discover false positives or negatives in this study. I would change this to present or absent.

**Discussion:**

- "CLE patients weren’t found in the sampled population" - they were excluded based on methods stated above.
- "Interestingly, one of our patients reported symptoms of numbness and facial sensory impairment, which indicates the involvement of sensory ganglia of the cranial nerves. Loss of taste and dry mouth were reported as the first manifestation of SLE in this patient. The serological result reported that the antinuclear antibody was present in a titer of 1/320, and the CT scan examination of the brain revealed that the patient had had a stroke. This may be attributed to the autoimmune autoantibodies directed against sensory ganglion":
  1. This belongs in results.
  2. How does the stroke, which I am assuming is an ischemic stroke in a specific area associated with the deficit, support antibodies against sensory ganglia? This is confusing and needs to be clarified by the authors. Did the patient have positive anti-phospholipid antibodies?
- "In the current study, oral candidiasis was observed in 41% of all the patients. Moreover, (74.3%) patients had oral lesions superinfected by *Candida*." - this was based on appearance and not culture/biopsy? I think this needs to be removed as this is not confirmed *Candida *according to the methods.

**Conclusion:**

- The link to cancer is not substantiated by current evidence and needs to be removed. I agree that more research in diverse settings is critical. Do the authors have a citation for the claim that research is only conducted in 1 in 10 countries? All research? Research on SLE? If no citation please make a more broad statement.

Is the work clearly and accurately presented and does it cite the current literature?

No

If applicable, is the statistical analysis and its interpretation appropriate?

Partly

Are all the source data underlying the results available to ensure full reproducibility?

Yes

Is the study design appropriate and is the work technically sound?

No

Are the conclusions drawn adequately supported by the results?

No

Are sufficient details of methods and analysis provided to allow replication by others?

No

Reviewer Expertise:

SLE, global health, pediatric rheumatology, genetics, translational research

I confirm that I have read this submission and believe that I have an appropriate level of expertise to state that I do not consider it to be of an acceptable scientific standard, for reasons outlined above.

## References

[1] : Oral mucosal lesions in systemic lupus erythematosus--a clinical, histopathological and immunopathological study. *J Rheumatol* .1984;11(1) :38-42 6366226

[2] : Oral lesions in systemic lupus erythematosus. Do ulcerative lesions represent a necrotizing vasculitis?. *J Am Acad Dermatol* .1992;27(3) : 10.1016/0190-9622(92)70204-s 389-94 10.1016/0190-9622(92)70204-s 1401272

[3] : Oral mucosal ulceration in systemic lupus erythematosus. *Arthritis Rheum* .21(1) : 10.1002/art.1780210110 58-61 10.1002/art.1780210110 623694

# Response

**Published:** 2022-01-06

**Authors:** Hager Saeed

**Affiliations:** october 6 university, Egypt

**Dear Laura,**

 Thanks for your valuable report, I appreciate all your efforts to write this constructive report. 

 I do my efforts to be concise in the paper as this paper is the summary of the thesis defense which is about 150 pages. Unfortunately, I couldn’t share the full thesis with you to learn more from your wide experience. 

**The clinical description of oral lesions, both type and location references**

           Burket’s Oral Medicine, 12th Edition - Michael Glick - Google Books. Accessed August 23, 2021. https://books.google.com.eg/books?hl=en&lr=&id=cBEqCAAAQBAJ&oi=fnd&pg=PR1&dq=burket%E2%80%99s+oral+medicine.&ots=hkfOb1399V&sig=GjVjm9Gg5OGxOsyAph5UwMfx_XQ&redir_esc=y#v=onepage&q=burket%E2%80%99s%20oral%20medicine.&f=false

 This reference was used in 

 •    Khatibi M, Shakoorpour AH, Jahromi ZM, Ahmadzadeh A. The prevalence of oral mucosal lesions and related factors in 188 patients with systemic lupus erythematosus. Lupus. 2012 Oct;21(12):1312–5.

 •    Hammoudeh M, Al-momani A, Sarakbi H, Chandra P, Hammoudeh S. Oral Manifestations of Systemic Lupus Erythematosus Patients in Qatar: A Pilot Study. International Journal of Rheumatology. 2018;2018:1-7. doi:10.1155/2018/6052326

 •    Hadidi KT El, Medhat BM, Baki NMA, Kafy HA, Abdelrahaman W, Yousri AY. Characteristics of systemic lupus erythematosus in a sample of the Egyptian population : a retrospective cohort of 1109 patients from a single center. 2018;(December 2017):1–9.

**Introduction:**

**•    "The prevalence of mucosal involvement in LE patients is debatable." - There are multiple reports on mucosal involvement in SLE. Authors should state there is a range based on population and cite the following: 1, 2, 3**

 Thank you for this valuable addition.

 •    " **The WHO considers the oral manifestations of LE as a widespread state associated with a significantly increased risk of cancer.” **–

  Thank you for this valuable addition. I removed this part.

 • **Inclusion criteria: Patients recently diagnosed with lupus erythematosus based on American College of Rheumatology (ACR) criteria. How did the authors define a recent diagnosis?**

 The Internal Medicine Department, Rheumatology Clinics in EL Qasr EL Ainy Hospital, Cairo University, has two clinics for lupus patients. Clinic One is only for new patient diagnosis, and Clinic Two is for treatment follow-up. New patients arrive at Clinic 1 in search of a diagnosis and are given Medical Record numbers. Clinic 1 was where all of the new patients were diagnosed. Patients with MRN who needed to be followed up on went to clinic 2.

 The research was carried out at Clinic No. 1. Only patients who were diagnosed immediately according to ACR criteria were included in the study. All of the patients in Clinic One had not previously received any lupus medication.

 • **Exclusion criteria - Patients who had received any previous therapy for lupus erythematosus. Authors should state treatment of naïve patients in the inclusion criteria.**

 Yes, the study was conducted in clinic number one, which is for new patients only.

 Only patients who were immediately diagnosed were included, as stated in the inclusion criteria. All of the patients in Clinic One were immediately diagnosed and had not previously received any lupus medication.

 In the thesis, we defined the drugs for lupus treatment as:

 Systemic Corticosteroids High doses (40 to60 mg/d of prednisone or prednisone equivalent) are used in patients with severe SLE. Doses of 10 mg/d or less are used for milder SLE for treatment of cutaneous and musculoskeletal symptoms not responding to other therapies. (Mehat et al., 2017)

 Immunosuppressants - Patients who are not responsive to anti-malarials or glucocorticoids should be considered for treatment with immunosuppressive agents for more severe manifestations of the disease (Bernknopf, 2015):

 •    Methotrexate. (MTX) is a folic acid analog which inhibits the enzyme dihydrofolate reductase and, as consequences of the proliferation of T cell populations (Bernknopf, 2015).

 •    Mycophenolate mofetil and mycophenolate sodium. Decreased activity of this enzyme affects proliferation of B and T lymphocytes induces apoptosis of activated T lymphocytes (Winkelmann, 2013).

 •    Azathioprine. is the prodrug of 6- mercaptopurine, Side effects include bone-marrow toxicity, gastrointestinal symptoms and hepatotoxicity (Winkelmann, 2013).

 •    Clofazimine. is an antibiotic with immunosuppressive and anti-inflammatory activity traditionally used in the treatment of leprosy (Winkelmann, 2013).

 BIOLOGIC AGENTS

 •    Intravenous immunoglobulin (IVIG). IVIG is the product of pooling immunoglobulin G (IgG) immunoglobulins extracted from donor blood (Winkelmann, 2013).

 •    Rituximab. Rituximab is a chimeric anti-CD20 monoclonal antibody that induces depletion of B cells through both antibody-dependent and independent pathways (Winkelmann, 2013).

 IMMUNOMODULATORS

 •    Dapsone - Dapsone is a sulfone that inhibits dihydrofolic acid synthesis and exhibits both antibiotic and anti-inflammatory properties.

 •    Thalidomide - The effects of thalidomide are attributed to the inhibition of TNF-alpha synthesis and UVB-induced keratinocyte apoptosis.

 •    Lenalidomide - Lenalidomide is a structural analog of thalidomide with more potent immune-modulatory effects and a lower risk of polyneuropathy (Winkelmann, 2013).

 • **"The diagnosis of oral candidiasis was made by curd-like patches on the tongue or other oral mucosal surfaces, the presence of classic pseudomembranous lesions characterized by a creamy white pseudomembrane" - was this confirmed by any culture or biopsy?**

According to Hopkins Lupus Cohort.

 The diagnosis of oral candidiasis was made by the presence of classic pseudomembranous lesions

 characterized by creamy white, curd-like patches

 on the tongue or on other oral mucosal surfaces. Oral candidiasis was defined at every visit by visual

 inspection of the oral cavity by one rheumatologist (Dr. Michelle Petri).

 Fangtham M, Magder LS, Petri MA. Oral candidiasis in systemic lupus erythematosus. Lupus. 2014;

**Results:**

**•    Table 1 should include an overall demographic data for all participants- age, sex, smoking, presence of oral lesions.**

  Thank you for this constructive addition.

 • **Table 1 should be Table 2.**

 Thank you for this constructive addition. I should back to the editor in this point.

 • **Figure 1 - would change to anatomical sites of oral lesions. How did the authors standardize the bounds of each lesion?**

  according to the methodology, we follow the mentioned references in the clinical description and clinical site.

        Burket’s Oral Medicine, 12th Edition - Michael Glick - Google Books. Accessed August 23, 2021. https://books.google.com.eg/books?hl=en&lr=&id=cBEqCAAAQBAJ&oi=fnd&pg=PR1&dq=burket%E2%80%99s+oral+medicine.&ots=hkfOb1399V&sig=GjVjm9Gg5OGxOsyAph5UwMfx_XQ&redir_esc=y#v=onepage&q=burket%E2%80%99s%20oral%20medicine.&f=false

 • **Figure 3: I am a bit confused about the inclusion of the data on the skin manifestations. Unless the authors have a specific hypothesis they would like to explore with this data, it seems out of place in this paper on oral lesions in SLE.**

 Thank you for this constructive addition. According to your direction, We removed this part.

**•    "The sampled population was classified into two groups. LE patients who had an oral manifestation as true positive oral lesions (TP) and LE patients without oral manifestation as true negative oral lesions (TN)." - I think this language is confusing, as they do not discover false positives or negatives in this study. I would change this to present or absent.**

 Thank you for this constructive addition. According to your direction, We amend it.

**Discussion:**

**•    "CLE patients weren’t found in the sampled population" - they were excluded based on methods stated above.**

  Thanks for your notification, but we didn’t exclude CLE. All the sampled patients had systemic involvement.

**•    "Interestingly, one of our patients reported symptoms of numbness and facial sensory impairment, which indicates the involvement of sensory ganglia of the cranial nerves. Loss of taste and dry mouth were reported as the first manifestation of SLE in this patient. The serological result reported that the antinuclear antibody was present in a titer of 1/320, and the CT scan examination of the brain revealed that the patient had had a stroke. This may be attributed to the autoimmune autoantibodies directed against sensory ganglion":**

 Thank you for this constructive addition, I removed the case.

 But, to clarify your doubts the patients was positive anti-phospholipid antibodies

 • **"In the current study, oral candidiasis was observed in 41% of all the patients. Moreover, (74.3%) patients had oral lesions superinfected by Candida." - this was based on appearance and not culture/biopsy? I think this needs to be removed as this is not confirmed Candida according to the methods.**

 Thank you for this constructive addition, I removed this part.

**Conclusion:**

**•    The link to cancer is not substantiated by current evidence and needs to be removed. I agree that more research in diverse settings is critical. Do the authors have a citation for the claim that research is only conducted in 1 in 10 countries? All research? Research on SLE? If no citation please make a more broad statement.**

 Thank you for this constructive addition, I amended this part.

 But to clarify your doubts,  this was mentioned by another reviewer as only 1/10 of all countries in the world assessed the prevalence of oral manifestation in lupus erythematosus.

# Response

**Published:** 2022-03-28

**Authors:** Hager Saeed

**Affiliations:** october 6 university, Egypt

I hope you're doing well. In accordance with your report, we have submitted the third version. 

 If you have any further questions, please let us know so we will make the necessary changes. 

 Thank you for the detailed instructions.

# Peer review: Reviewer response for version 1

**DOI:** 10.5256/f1000research.58897.r95501

**Published:** 2021-10-12

**Authors:** Syahrul Sazliyana Shaharir

**Affiliations:** 1 Department of Internal Medicine, Rheumatology Unit, Universiti Kebangsaan Malaysia Medical Center, Kuala Lumpur, Malaysia

This was an interesting study but I have a few queries:

1. It was mentioned that Egyptian patients recently diagnosed with lupus erythematosus were included in the study. What was the definition of recently diagnosed? Within 3 months of diagnosis or at the onset of diagnosis?
2. "Exclusion criteria: Patients who had received any previous therapy for lupus erythematosus". So were all patients treatment-naïve?
3. What were the example of drugs that may become confounder of oral lesions?
4. What were the questionnaire questions? Kindly elaborate more what were the questions asked to the patients as these data were not presented in the Results section.
5. Results: the data presented was very minimal. In the methodology it was mentioned that a full history was obtained through an interview. In addition, subjects were also given a set of questionnaire (in which the content of the questions were not clear). The results did not elaborate the “full history” that was obtained. There was no mentioned of other clinical manifestations of SLE apart from skin manifestation. And no data on the background treatment or medications, if present.
6. In discussion, the most likely reason for no association between oral lesion with smoking status was due to very small sample size in the smoking arm.
7. It was stated in the discussion that oral candidiasis was observed in 41% of all the patients. Moreover, (74.3%) patients had oral lesions superinfected by *Candida*. This was not mentioned in the Methodology and Results, but what was the difference between oral candidiasis and oral lesions superinfected by *Candida*? How was the diagnosis made to differentiate the 2 conditions?
8. The discussion was too long but the results were too brief. Authors should focus and discuss the results that answer the primary and secondary objectives of the study. Should exclude discussion about the nerve involvement as it was not part of the study objectives.

Is the work clearly and accurately presented and does it cite the current literature?

No

If applicable, is the statistical analysis and its interpretation appropriate?

Yes

Are all the source data underlying the results available to ensure full reproducibility?

Partly

Is the study design appropriate and is the work technically sound?

Yes

Are the conclusions drawn adequately supported by the results?

Yes

Are sufficient details of methods and analysis provided to allow replication by others?

No

Reviewer Expertise:

SLE

I confirm that I have read this submission and believe that I have an appropriate level of expertise to state that I do not consider it to be of an acceptable scientific standard, for reasons outlined above.

# Response

**Published:** 2021-12-19

**Authors:** Hager Saeed

**Affiliations:** october 6 university, Egypt

***It was mentioned that Egyptian patients recently diagnosed with lupus erythematosus were included in the study. What was the definition of recently diagnosed? Within 3 months of diagnosis or at the onset of diagnosis?***

 The Internal Medicine Department, Rheumatology Clinics in EL Qasr EL Ainy Hospital, Cairo University, has two clinics for lupus patients. Clinic One is only for new patient diagnosis, and Clinic Two is for treatment follow-up. New patients arrive at Clinic 1 in search of a diagnosis and are given Medical Record numbers. Clinic 1 was where all of the new patients were diagnosed. Patients with MRN who needed to be followed up on went to clinic 2.

 The research was carried out at Clinic No. 1. Only patients who were diagnosed immediately according to ACR criteria were included in the study. All of the patients in Clinic One had not previously received any lupus medication.

***"Exclusion criteria: Patients who had received any previous therapy for lupus erythematosus". So were all patients treatment-naïve?***

 Yes, the study was conducted in clinic number one, which is for new patients only.

 Only patients who were immediately diagnosed were included, as stated in the inclusion criteria. All of the patients in Clinic One was immediately diagnosed and had not previously received any lupus medication.

 In the thesis, we defined the drugs for lupus treatment as:

 Systemic Corticosteroids High doses (40 to60 mg/d of prednisone or prednisone equivalent) are used in patients with severe SLE. Doses of 10 mg/d or less are used for milder SLE for treatment of cutaneous and musculoskeletal symptoms not responding to other therapies. (Mehat *et al.*, 2017)

 Immunosuppressants - Patients who are not responsive to anti-malarials or glucocorticoids should be considered for treatment with immunosuppressive agents for more severe manifestations of the disease (Bernknopf, 2015):

- Methotrexate. (MTX) is a folic acid analog which inhibits the enzyme dihydrofolate reductase and, as consequences of the proliferation of T cell populations (Bernknopf, 2015).
- Mycophenolate mofetil and mycophenolate sodium. Decreased activity of this enzyme affects proliferation of B and T lymphocytes induces apoptosis of activated T lymphocytes (Winkelmann, 2013).
- Azathioprine. is the prodrug of 6- mercaptopurine, Side effects include bone-marrow toxicity, gastrointestinal symptoms and hepatotoxicity (Winkelmann, 2013).
- Clofazimine. is an antibiotic with immunosuppressive and anti-inflammatory activity traditionally used in the treatment of leprosy (Winkelmann, 2013).

BIOLOGIC AGENTS

- Intravenous immunoglobulin (IVIG). IVIG is the product of pooling immunoglobulin G (IgG) immunoglobulins extracted from donor blood (Winkelmann, 2013).
- Rituximab. Rituximab is a chimeric anti-CD20 monoclonal antibody that induces depletion of B cells through both antibody-dependent and independent pathways (Winkelmann, 2013).

IMMUNOMODULATORS

- Dapsone - Dapsone is a sulfone that inhibits dihydrofolic acid synthesis and exhibits both antibiotic and anti-inflammatory properties.
- Thalidomide - The effects of thalidomide are attributed to the inhibition of TNF-alpha synthesis and UVB-induced keratinocyte apoptosis.
- Lenalidomide - Lenalidomide is a structural analog of thalidomide with more potent immune-modulatory effects and a lower risk of polyneuropathy (Winkelmann, 2013).

***What were the example of drugs that may become confounders of oral lesions?***

 This point aims to eliminate the confounders. For example, some types of antibiotics cause changes in the microbial flora of the oral cavity and increase candida infection.

 As mentioned in the following references some drugs induce oral lesions:

- Oral ulcerations due to drug medications
- An Update on Drug-induced Oral Reactions
- A Review of Drug-Induced Oral Reaction
- Drug-Induced Oral Reactions

***What were the questionnaire questions? **Kindly elaborate more what were the questions asked to the patients as these data were not presented in the Results section.***

 The patient enters Clinic One (new patient clinic). The patient opens a file (include all the Demographical data) **.** The specialized nurse record the vital signs and the history of the patients. After that the patient entered the doctor's clinic and the patient's full history was taken. The patients were examined initially by a rheumatologist and were later be scheduled for an appointment with the same dentist at the same institution, for an oral and dental examination. The study includes a group of patients with a confirmed diagnosis of LE who presented to the Internal Medicine department, rheumatology clinic in EL Qasr EL Ainy hospital - Cairo University.

 Diagnosis of LE was established based on the criteria established by the American College of Rheumatology based on tests that confirm LE diagnosis (ANA) only was included in the study.

***Results: the data presented was very minimal. In the methodology it was mentioned that a full history was obtained through an interview. In addition, subjects were also given a set of questionnaire (in which the content of the questions were not clear). The results did not elaborate the “full history” that was obtained. There was no mention of other clinical manifestations of SLE apart from skin manifestation. And no data on the background treatment or medications, if present*** **.**

 Full history was obtained by the rheumatologist to diagnose the patients. The rheumatologist documented the history and the requested investigation in the patient file.

 In my role as a dentist, if the ANA test is positive I scheduled an appointment, for an oral and dental examination at the same institution. The study focused only on the outcomes that’s why the results demonstrate the demographic and outcomes only.

 Outcomes:

- Primary outcome: Prevalence of intraoral manifestations. As ulcer (a defect in the epithelium in the form of a depressed lesion), erythema, white plaque (a solid raised lesion greater than 1 cm in diameter), spots or white striae with a radiating orientation.
- Secondary outcome: Extraoral and perioral findings. malar rash, photosensitive dermatitis, generalized maculopapular rash, discoid rash, subacute cutaneous lupus erythematosus (SCLE), lupus profundus, erythema multiforme.

***In discussion, the most likely reason for no association between oral lesion with smoking status was due to very small sample size in the smoking arm.***

 Yes, I agree with you. I wrote this paragraph in the thesis:

 Smoking cessation is recommended in controlling CLE symptoms (Chang *et al*., 2016). Studies also report the decrease of chloroquine efficacy in smokers, due to the effect of tobacco on cytochrome P450, which enzymatic system is responsible for the metabolism of this drug. In addition, smoking is related to other risk factors that also influence treatment adherence (Moura *et al.*, 2014).

 No significant differences were reported in some habits such as smoking or flossing frequency. Studies have reported that SLE patients have a reduced oral health-related quality of life (HRQoL) comparable to their counterparts with severe medical diseases, such as AIDS, diabetes and rheumatoid arthritis (Corrêa *et al*., 2018).

 In the multivariate analysis, being a current smoker was associated with the presence of active rash. No clear association was seen between mucosal ulcers and smoking across the various smoking groups. No clear association was seen between smoking and the presence of the ACR criteria of malar rash or mucosal ulcers (Bourré *et al*. 2013).

 In contrast to that, Chang reported in a prospective cohort study of CLE patients indicated that the greater disease severity and the worse quality of life measurements in current smokers (Chang *et al., *2016). Smoke activates metalloproteinases, that damage the tissue, and cytokines such as interleukin- 6, an important marker of inflammation in lupus (Moura *et al.,* 2014). 

***It was stated in the discussion that oral candidiasis was observed in 41% of all the patients. Moreover, (74.3%) patients had oral lesions superinfected by Candida. This was not mentioned in the Methodology and Results, but what was the difference between oral candidiasis and oral lesions superinfected by Candida? How was the diagnosis made to differentiate the 2 conditions?***

 The diagnosis of oral candidiasis was made by curd-like patches on the tongue or on other oral mucosal surfaces, the presence of classic pseudomembranous lesions characterized by creamy white pseudomembrane (Fangtham *et al.,* 2014). All the oral candidiasis can be rubbed off by swap. In case of white lesions, the candida will be rubbed off but the lesion will not be removed. In this study, we found that 41% of all the sampled patients (189) had oral candidiasis. Moreover, we found that the prevalence of oral candidiasis (seventy-eight (87) out of 105) was (74.3%).

 Oral candidosis (OC) is subdivided into primary and secondary. Secondary infections are superimposed on other diseases of the oral mucous membranes, such as oral lichen planus (OLP), a chronic inflammatory disease.

 Oral Mucosal T-Cell Responses to *C. Albicans*: Fungal infection is one of variant well-known opportunistic infections in patients with SLE. Candida is the commonest opportunistic fungal infection recognized. Host factors such as decreased salivary flow rate or smoking are associated with the raised oral carriage rate of Candida. SLE can significantly decrease the salivary flow rate compared to healthy individuals (Fangtham *et al*., 2014).

 Resistance to the fungal infections caused by *C. Albicans* needs coordinated action of the innate and adaptive immune responses, during which the activation of systems activate the secretion of multiple of primary cytokines and expression of co-stimulatory molecules. (McIntyre, 2001)

 See diagram here:

*https://f1000researchdata.s3.amazonaws.com/linked/396723.ReviewforLW.PNG*

 Schematic representation of the important interactions between the immune system of the host, and bacterial microbiota and *C. Albicans* in the GI tract ****(Cottier *et al*., 2012).

 TNF-α is liberated by macrophages during the early phase of the inflammatory response to fungus attracts and activates neutrophils to become antifungal effectors. The production of IL-4 is dependent on the amount of fungus present in the infection site. IL-10 was first described as a cytokine that had potent anti-inflammatory activity (McIntyre, 2001).

 As the reason of the consequent secretion of pro-inflammatory cytokines (IL-6, IL-8, IL-10, IL-12, TNF-α) in LE, these cytokines act as a potent anti-inflammatory and this affects immune regulation of antifungal effector. This proves the correlation between candida and LE (Ferretti *et al.*, 2016).

 A study conducted in USA reported that oral candidiasis can be found in conjunction with occult esophageal infection. This indicates that, disseminated Candida infection can originate from the oral cavity (Fangtham *et al*., 2014). This may indicate the presence of xerostomia in LE increases susceptibility to oral infections, mainly oral candidiasis (Fangtham *et al.*, 2014).

 In a study conducted in Japan, all patients with xerostomia showed the pathophysiology of atrophicans oral candidiasis. In addition, the prevalence of oral candidiasis was significantly higher in patients with xerostomia than in controls (Shinozaki, S., *et al. *2012).

 Also, Torres had conducted a study to assess the correlation between salivary flow rates and *Candida* counts it was found that the frequency of *Candida* colonization was (67.9%) (Torres *et al*., 2002).

 In addition to that, SLE disease activity is a risk factor for *Candida* infection since, high SLE disease activity is associated with invasive fungal infections (Fangtham *et al.*, 2014).

 Interestingly, the prevalence of oral candida in African patient may be attributed to ethnicity. Whereas, African-American ethnicity had a higher risk of oral candidiasis (Fangtham *et al.*, 2014). On the other hand, opportunistic infections, particularly *Candida *infections, are more common in patients with SLE because of altered immune status (Cojocaru *et al*., 2011).

***The discussion was too long but the results were too brief. Authors should focus and discuss the results that answer the primary and secondary objectives of the study. Should exclude discussion about the nerve involvement as it was not part of the study objectives.***

 Yes, you have a valid point of view. To clarify your doubts about the results. According to the author's guide, I couldn’t add all the tables and figures.  

 In terms of nerve involvement, this case explains that the first symptom of nerve involvement could be numbness. Among the sampled patients, one of the present study patients reported symptoms from involvement of sensory ganglia of multiple cranial nerves such as facial sensory impairment and facial numbness (gasserian ganglion of trigeminal nerve), loss of taste (geniculate ganglion of facial nerve). Additional contributing factor to the loss of taste could be dry mouth. This was the first manifestation of SLE. The serological result reported that antinuclear antibody was present in a titre of 1/320. The CT scan examination of the brain reported that there was a stroke.

 Interestingly, the first trigeminal neuropathy (TN) case in SLE patients was reported in 1971 where TN has been reported as the only neurological manifestation of the SLE among 2 cases. (Ashworth *et al*., 1971). Also, in 1990 Hagen *et al*. reported 2 cases of TN in SLE among 81 studied subjects. More recently, Kumar *et al.* (2017) reported one case of TN during the course of SLE in 35 years African American woman patient in USA. Finally, Lefter *et al.* (2020) reported a case with acute severe sensory ganglionopathy in 24 years man through the course of SLE. This proves that TN can be caused by autoimmune response and immune complex deposition in the vessels. The diagnosis of TN is based on the characteristic clinical picture, which is considered the key clinical features and physical examination showing no clinical evidence of neurological deficit or mild sensory impairment in trigeminal nerve distribution (Kumar *et al.*, 2017).

 Furthermore, Facial numbness, paresthesia, dysesthesia, and pain have been reported most frequently; TN may be the first feature of SLE or might follow the onset of the disease, usually developing slowly over the course of the illness (Hagen, 1990). Autoantibodies against the ganglionic acetylcholine receptor, reported in the serum of 12.5% SLE patients, might play a role in the autonomic disturbance of these patients (Kumar *et al.,* 2017). The most important thing, that tongue stiffness can be the initial symptom of an autoimmune disease (Rajevac *et al.*, 2020).
